# Microcapsule encapsulated with leuco dye as a visual sensor for concrete damage indication *via* color variation

**DOI:** 10.1039/c9ra09492j

**Published:** 2020-01-08

**Authors:** Yao Li, Qing Wang, Xu Zheng, Yunfeng Li, Jinjin Luan

**Affiliations:** Institute of NanoEngineering, College of Civil Engineering and Architecture, Shandong University of Science and Technology Qingdao 266590 China qwang@sdust.edu.cn

## Abstract

A microcapsule-type visualization sensor for concrete structural damage indication is proposed in this article. Crystal violet lactone, as damage indicator, was microencapsulated within poly(methyl methacrylate) to synthesize the sensor. The successful encapsulation was confirmed by Fourier transform infrared spectrometry. Microcapsules of different diameters and size distributions were obtained by varied stirring speeds. The fabricated microcapsules were embedded into a polymer coating to accomplish the damage indication. When cracks propagated in the coating, the crystal violet lactone in leuco form was released from the ruptured microcapsules. Due to reacting with silicon dioxide in concrete, the released crystal violet lactone turned blue and highlighted the damaged area. It was verified that the visualization performance of the sensor showed good durability in both dry and wet conditions. The proposed microcapsule-type visualization sensor has advantages of easy fabrication, high indication stability, and no special equipment requirements, which will reduce the complexity of concrete structural health monitoring significantly.

## Abstract 2

A visual microcapsule sensor for concrete damage detection by color variation without the requirement for additional activators.

## Graphical abstract

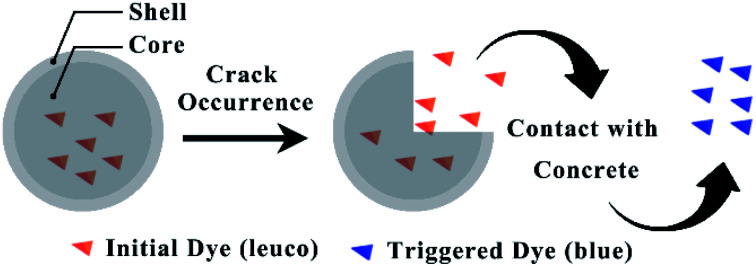

## 1. Introduction

All cementitious materials, especially concrete, are susceptible to forming micro-cracks, which eventually lead to structure failure.^1^ Therefore, crack monitoring for concrete structures is critical to improve lifespan and stability. Structural health monitoring (SHM) technologies to monitor damage are applied extensively throughout civil engineering.^2,3^ Since micro-cracks are hardly visible to the naked eye, specialized instruments such as optic fiber sensors,^4,5^ piezoelectric sensors^6–9^ and data processing equipment are required for traditional SHM technology.

Novel material-based smart sensors provide new approaches to overcome these problems. A series of visual damage strategies based on visualization materials had been developed for damage indication.^10–14^ Dye-filled hollow glass fiber^15–17^ was used to indicate mechanic damages for polymers, but it cannot be applied to the surfaces of complex structures because of its slender shape. The microcapsule-based visualization sensor solves these problems better.^18–25^ Microcapsules can be easily embedded in a polymer film because of its micro-scale. And varied formation methods make it suitable for a variety of surfaces. Recently, a microcapsule-type fluorescence probe system had been proposed for damage indication of cementitious materials.^23^ However, indicating cracks using fluorescence is ineffective because microcapsules by themselves are fluorescent, which will seriously interfere with crack detection. Because fluorescence cannot be captured by the naked eye in daylight, specific wavelengths of light were required for fluorescence observation.

**Fig. 1:**
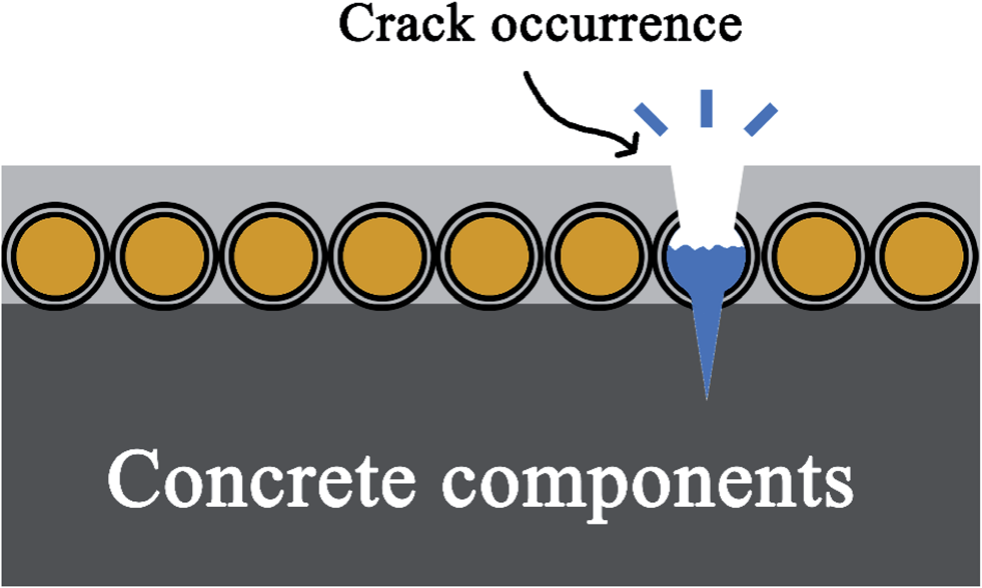
Schematic diagram of damage indication. When micro-cracks occur and propagate in the coating, CVL will be released from ruptured microcapsules and highlights the crack in blue.

To solve these problems, a microcapsule-based damage indication strategy for concrete structures was proposed in this study. For damage indication, crystal violet lactone (CVL) containing microcapsules were applied to the surface of concrete block. As shown in Fig. 1, when micro-cracks occur and propagate in the coating, CVL will be released from ruptured microcapsules and highlights the crack in blue. In addition, no color developer or activation is required for damage indication.

In this paper, microcapsules for damage indication of concrete structure were fabricated by solvent evaporation. The morphology and successful encapsulation were confirmed by scanning electron microscope (SEM) and Fourier transform infrared (FT-IR) spectrometer, respectively. The relationship between microcapsule diameter and the stirring speed was investigated by a laser particle size analyzer. Finally, the microcapsules were coated on a concrete block to test the damage indication performance and indication durability.

## 2. Experiments

### 2.1. Materials

CVL (≥98%, Cool Chemistry, China), poly(methyl methacrylate) (PMMA, 100 mesh, Zhongxin Plastic, China), dichloromethane (>99.5%, Chron Chemicals, China), phenyl acetate (99%, Macklin, China), polyvinyl alcohol (PVA, 1788, Usolf, China).

### 2.2. Instruments

The surface morphology of microcapsules was characterized by scanning electron microscopy (APREO, FEI, USA). The successful encapsulation of microcapsules was confirmed by Fourier transform infrared spectroscopy (Nicolet iS50 FT-IR, Thermo Fisher Scientific, USA). The effect of stirring speed on size distribution and main diameter was investigated by a laser particle size analyzer (Mastersizer 2000E, UK). The drying microcapsules were obtained by a vacuum drying oven (Lange, IPC-25, China). A magnetic stirrer was used to form the microcapsules (Yuhua, Hwet, China).

### 2.3. Microcapsules

**Fig. 2:**
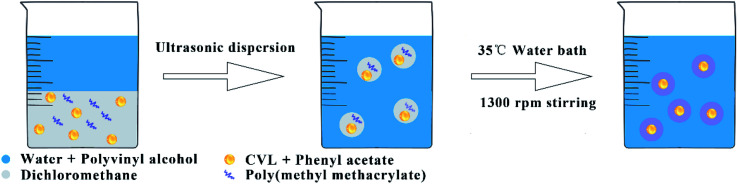
Scheme of fabrication progress of microcapsules.

Fig. 2 shows the fabrication progress of microcapsules. The microcapsules were fabricated by the solvent evaporation method. First, 1 g of PMMA was dissolved in 30 g of dichloromethane and 0.25 g of CVL was dissolved in 2 g of phenyl acetate in different containers. The two solutions were mixed as oil phase and ultrasonic dispersed for two minutes by an ultrasonic disperser. Then, 1.6 g of PVA as emulsion stabilizer was slowly dissolved in 78.4 ml of 80 °C deionized water at 500 rpm by a magnetic stirrer, cooled to room temperature as water phase. The oil phase was added into the water phase slowly at room temperature under a 1500 rpm stirring by a magnetic stirrer. The resulting emulsion was stirred for 5 hours in a 35 °C water bath to evaporate the dichloromethane to form microcapsule dispersion. The dispersion was washed 3 times with deionized water. Remove the supernatant after standing for 24 hours to obtain the suspension of microcapsules. Dry microcapsules were obtained at 50 °C for 10 hours by a vacuum oven.

## 3. Results and discussion

### 3.1. Characterization of microcapsules

**Fig. 3:**
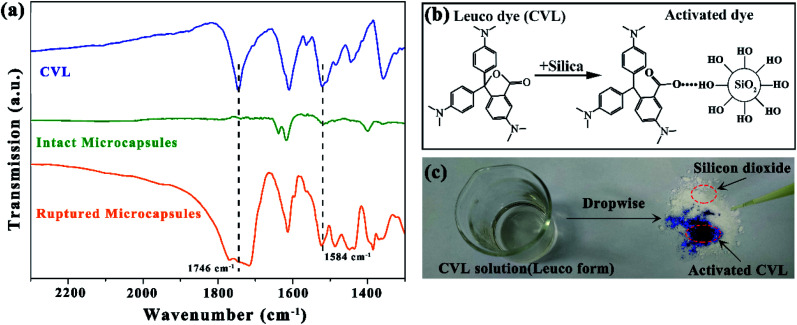
(a) FT-IR spectra of CVL, intact microcapsules and ruptured microcapsules, (b) schematic of CVL color change and (c) photograph of CVL before and after contacting with silicon dioxide.

The FT-IR spectra of CVL, intact microcapsules and ruptured microcapsules are shown in Fig. 3a. The successful encapsulation was confirmed by a FT-IR spectrometer. An agate mortar was used to rupture the microcapsules. The shell and core materials of the ruptured microcapsules were mixed together with potassium bromide to prepare the pellet. As Fig. 3a shows, in the spectra of ruptured microcapsules, the characteristic absorbance peak at 1746 cm^−1^ is assigned to the C=O stretching vibration in CVL,^26,27^ which verified the successful encapsulation of microcapsules. During the grinding process of microcapsules, CVL reacts with hydroxyl groups on silicon dioxide in the agate mortar as Fig. 3b, which protonates CVL and raises a new peak at 1584 cm^−1^ because of the opening of the lactone ring.^27^ In the spectra of intact microcapsules, a weak peak also appears at 1584 cm^−1^, because during fabrication, trace amounts of phenyl acetate was dissolved in water and hydrolyzed to acetic acid and phenol. And the lactone ring of CVL can be opened by weak acid or proton donor,^28,29^ thus trace amounts of CVL with already opened lactone ring generates. During preparation of the pellet, few microcapsules are ruptured by pressure, leading to the weak peak at 1584 cm^−1^ in the spectra of intact microcapsules. But this slight color changing cannot be captured by the naked eye. The color changing after colorless CVL reacts with pure silicon dioxide is shown in Fig. 3c. After mixed with pure silicon dioxide, the colorless CVL turns to a strong blue color, which confirms the color reaction between leuco CVL and silicon dioxide.^29^

**Fig. 4:**
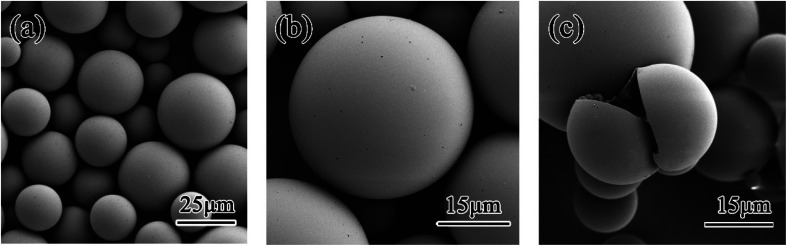
SEM images of (a and b) intact microcapsules and (c) ruptured microcapsules.

The SEM images of the dry microcapsules are shown in Fig. 4. The microcapsules were fabricated under 1500 rpm stirring. It can be seen from Fig. 4a and b, microcapsules had a uniform particle size distribution, mainly are 25 ± 10 μm, and formed a regular spherical surface. As shown in Fig. 4b, no adhesion and agglomeration occurred among microcapsules, which will benefit the dispersion of microcapsules in the coating. A sharp razor was used to make a cracked microcapsule. The shell structure of the cracked microcapsule can be seen from Fig. 4c.

### 3.2. Diameter distribution of the microcapsule

**Fig. 5:**
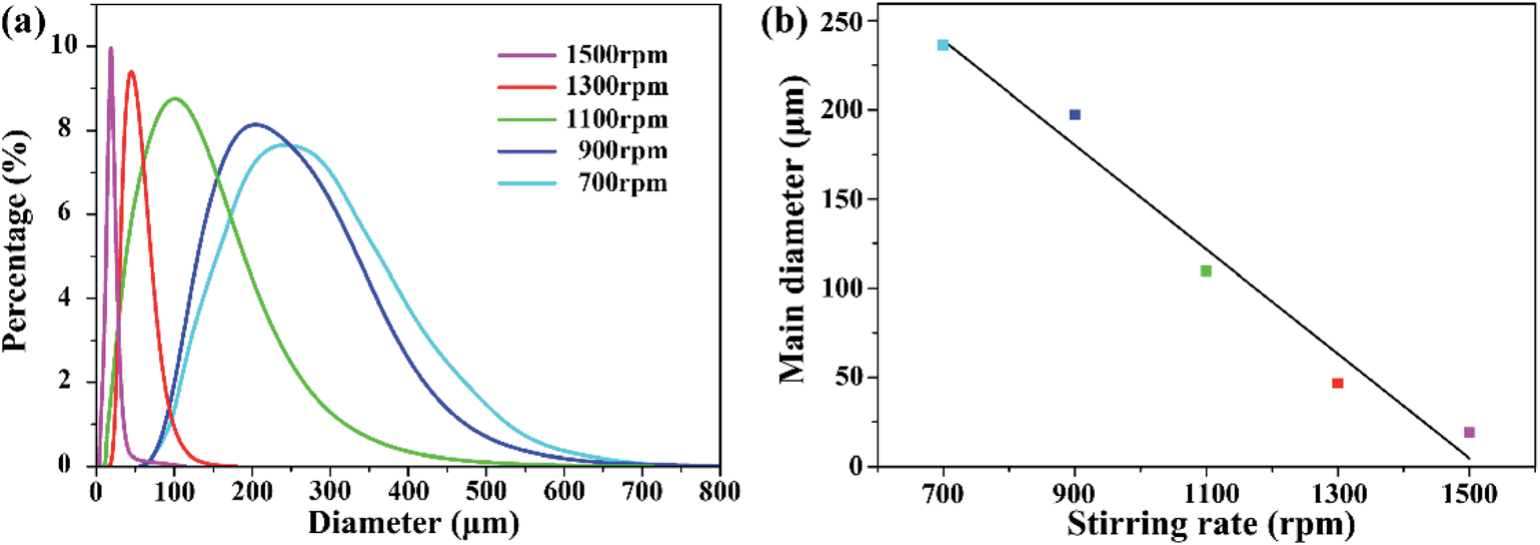
Effect of stirring speed on size distribution and main diameter. (a) Relationship between size distribution and stirring speed. (b) Main diameter at different stirring speeds.

The effect of stirring speed on size distribution and main diameter is shown in Fig. 5. The stirring speed was set from 700 to 1500 rpm while keeping the material ratio unchanged. Smooth spherical microcapsules were fabricated at 700–1500 rpm. As can be seen in Fig. 5a, changing the stirring speed will have a significant impact on the size distribution of the microcapsules. The size distribution of the microcapsules narrows as the stirring speed increased. Fig. 5b shows microcapsule diameter that accounts for the most proportion at different stirring speeds. The main diameter of microcapsules decreases as stirring speed increased, which is substantially linear. The increasing stirring speed provided larger shearing force and reduced the particle diameter of the oil phase droplets which can be stably present. During evaporation, PMMA attached to the surface of the oil droplets, formed smaller diameter and a narrower particle size distribution.

### 3.3. Visualization performance

**Fig. 6:**
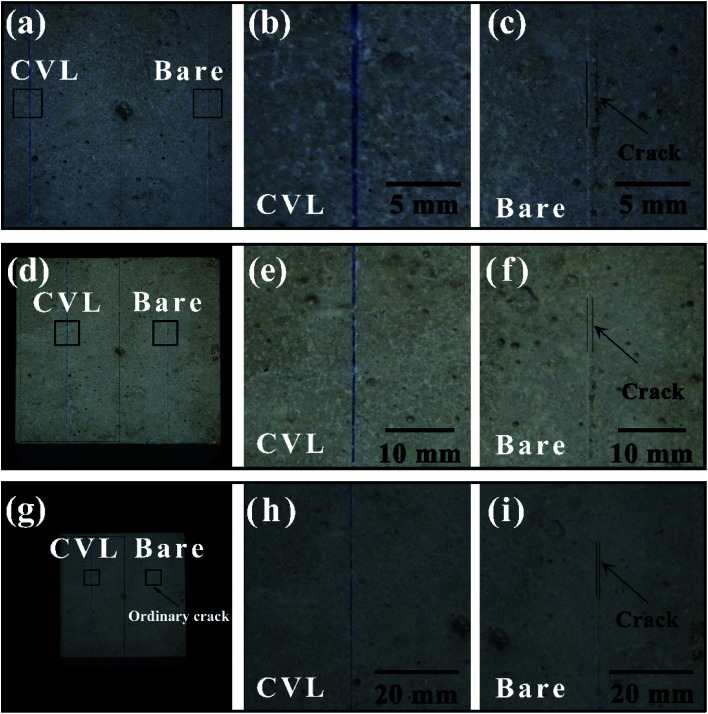
Photographs of cracks in microcapsule-containing coatings on concrete block surface at the distances of 20 cm (a) and enlarged view of crack with microcapsules (b)/without microcapsules (c), 50 cm (d) and enlarged view of crack with microcapsules (e)/without microcapsules (f), 1 m (g) and enlarged view of crack with microcapsules (h)/without microcapsules (i).

As shown in Fig. 6, the evaluation of the visualization performance of the visualization sensor is conducted for a concrete block. The concrete block (100 mm × 100 mm × 100 mm) was prepared by mixing stones, river sand, cement and water together. The block was cured for 28 days in a curing box after hardening for 24 hours. Since the good film-forming property of PVA, the suspension of microcapsules (left part) and pure PVA coating without microcapsules (right part) is brushed directly to the concrete surface. The coating was cut with a razor blade to form micro-cracks. Photographs were taken at distances of 20 cm, 50 cm, 1 m, as shown in Fig. 6a, d and g. It can be found that, comparing with the side without microcapsules in Fig. 6c, f, and i, and a highly visible blue color occurs in the left injury site which is coated microcapsules in Fig. 6b, e, and h. And the crack on the side without microcapsules is hardly visible at the distance of 1 m (Fig. 6g–i).

### 3.4. Visualization durability

**Fig. 7:**
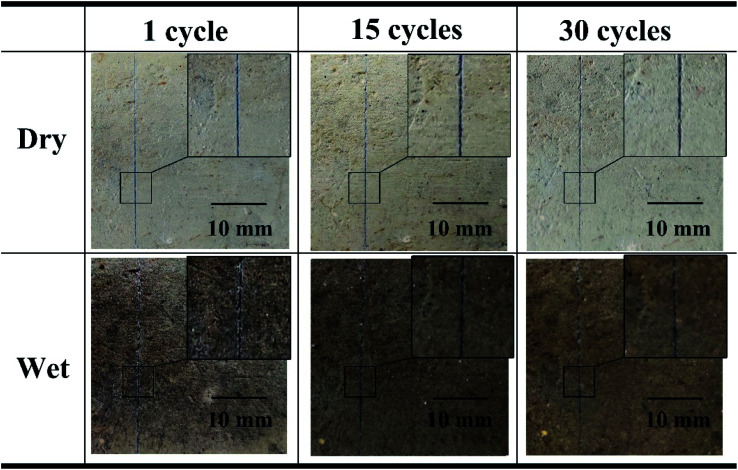
Visualization performance of microcapsule-type visualization sensor after different dry and wet cycles.

The durability of the coating in wet and dry environment is evaluated by the dry and wet cycle as shown in Fig. 7. First, cracks on the coating were manufactured by a razor. The concrete block covered with the microcapsule coating was air-dried for 30 minutes in the sun, and then placed in a box containing 20 ± 5 °C of water, the water was 30 mm above the upper surface of the concrete. After soaking for 5 min, drying for 30 minutes in sunlight for one cycle. It can be seen that damaged microcapsules produced a distinct blue contrast in both dry and wet conditions. After 30 times of cycles, as can be seen from the detailed image of the enlarged part, the blue color shows a good durability. The damage indication effect can be maintained after drying.

## 4. Conclusions

In this paper, the CVL containing microcapsule-type visualization sensor was successfully encapsulated by solvent evaporation. After confirmation of the successful encapsulation, the potential application and damage indication performance on concrete structure of the sensor were demonstrated. The visualization performance of the sensor showed good stability in the durability testing experiment. In addition, the proposed microcapsule-type visualization sensor has advantages of easy fabrication, high indication durability, and none special equipment requiring, which will reduce the costing in concrete structure health monitoring significantly.

## Conflicts of interest

The authors declare no competing financial interest.

## Supplementary Material
